# Affective Outcomes of Membership in a Sport Fan Community

**DOI:** 10.3389/fpsyg.2020.00881

**Published:** 2020-05-08

**Authors:** Brandon Mastromartino, James J. Zhang

**Affiliations:** Department of Kinesiology, International Center for Sport Management, University of Georgia, Athens, GA, United States

**Keywords:** spot fans, consumer behavior, fan communities, social capital, affective outcomes

## Abstract

In this mini-review, we shed light on the important, yet under researched topic area in sport management – understanding the role that emotion plays for members of sport fan communities. Much research has been done on the relationship between sport organization and fan; yet, far more needs to be discussed on how the sense of community fans feel lead to affective outcomes and consumption behaviors. With an understanding of the affective outcomes resulting from a connection between fans, sport organizations can use the knowledge to develop promotional procedures and nurture their fan community in an effort to grow their fan base and elevate consumption behavior. The aim of this mini-review is to (a) draw attention to the value of sport fan communities and (b) highlight areas in which sport organizations can build marketing strategies. As it is necessary for sport organizations to better understand the role emotion plays in the consumer behavior of their fans, more attention needs to be paid in understanding the affective benefits of membership in a sport fan community.

## Introduction

The behaviors of sport fans have been a widely studied concept in sport management research. There has been extensive research because fans are the lifeblood for sport organizations and it is important for sport organizations to understand fan consumer behaviors in order to better fit their needs and generate more profits (Wann and James, 2019). Sport fans reinforce identification with a team by engaging in supportive and repetitive consumption behaviors such as attending live events, purchasing team merchandise, and watching games on TV or through Internet streaming services (Mastromartino et al., 2018). Sport fans are different than fans of other products such as car or clothing brands due to a unique emotional attachment an individual has with their sports teams. Wakefield (2007) highlights this attachment:

Highly identified fans will internalize or adopt the team or player’s attitudes and behaviors as their own. If you are highly identified with a team, you feel good when the team wins, and bad when the team loses. You believe the team is a representation of who you are to yourself and to others. You practically feel as though you are part of the team (p. 37).

For a sport organization, it is more efficient to develop and maintain a diehard lifelong fan as opposed to constantly finding ways to engage new fans. Sport organizations are seeking to compete in the international marketplace, now more so than ever before. As the domestic market becomes saturated, sport organizations in North America are looking to build a global consumer base for their products (Zhang et al., 2014). This has led sport managers to believe that potential for long term growth can be accomplished through the global marketplace (Walker and Tehrani, 2001). For example, it is already evident in the National Basketball Association (NBA) with its development of international exhibition games and also programs such as Basketball Without Boarders. With new technologies, fan communities are stretching geographic boundaries, and with an understanding of how an individual connects with a fan community and the affective outcomes of that membership, sport organizations can expand their fan community across the globe.

Previous studies generally conclude that identification with a fan community is achieved when one feels a sense of connection with other members of the community and a sense of difference from those not in the community (Muniz and O’Guinn, 2001; Keller, 2003; Mastromartino et al., 2019b). Various elements of sport fan communities have been discussed in previous studies; however, they have mostly focused on behavioral outcomes, not psychological ones. For example, Yoshida et al. (2015) examined fan community identification of Japanese soccer and baseball fans. They found that identification and membership with the fan community had positive effects on team brand equity which resulted in increased fan community engagement, customized product use, member responsibility, and positive word of mouth. Grant et al. (2011) examined fan communities of newly formed professional sports teams in New Zealand to identify factors that contribute to building a successful brand community from scratch. They found that marketing strategies from the organization did not often match up with the markers of brand community and did not initiate marketing strategies to encourage the development of a brand community. Lastly, Mastromartino et al. (2019a) conceptualize a model of community identification where affective benefits are one of the perceived benefits of membership in a sport fan community. Previous research has hypothesized ideas regarding sport fan communities, but there have been no specific investigations into the role emotions play in sport fan communities. This highlights the need for more research to be done for organizations to better understand the affective outcomes of membership of fan communities and utilize them when formulating a marketing mix, which is particularly needed when attempting to lessen the impact of a lack of team history or success on fan identification and consumption behavior. This paper aims to examine the affective outcomes of membership in a sport fan community for the individual fan. Although there may be additional benefits on the group or societal level such as increases in national pride (Hallmann et al., 2013; Elling et al., 2014), these implications are outside the scope of this paper and should be examined in future research.

## Social Capital Theory

Social capital is the theoretical underpinning of understanding the psychological benefits one receives from membership in a sport fan community. Social capital can be defined as “resources embedded in a social structure which are accessed and/or mobilized in purposive actions” (Lin et al., 2001). This highlights that membership in a community has a value to each member and that value can be accessed by participating in that community. The value, or benefits, members gain vary from individual to individual, but can include an increase in self-esteem and sense of belonging (Wann et al., 2011). Sport fans participate in the community in many ways such as going to games, becoming in involved in social media discussion, or recruiting others to become fans. This type of participation increases a fan’s connection to the community, allowing access to more resources and benefits for the individual (Phua, 2012). Following this line of theory, the more one participates and the more connections one makes in the community, and the better off they will be emotionally (Nicholson and Hoye, 2008). A unique element of social capital is that it operates at various levels of a social structure, such as a sport fan community. It functions on the individual level for personal social capital gain, or it can be accessed to serve more general communal or societal needs (Coleman, 1988; Putnam, 1995). To oversimplify, The Beatles song The End states “And in the end, the love you take, is equal to the love you make.” This implies there is certain value to contributing to the community, which is social capital. Kadushin (2012) notes there are two types of social capital in communities: social capital investment and individual social capital. Social capital investment is what one puts into their community, such as volunteer work, and leadership roles can increase involvement among other community members or improvement in the community such as lower crime rates and economic growth. Individual social capital is what impacts well-being on the individual level, such as an increased sense of wellness or self-esteem. However, these are not mutually exclusive consequences as an improved and active community can contribute to increases in personal well-being as well. The types of outcomes each individual experiences will vary from person to person some members may receive parts of some, or parts of none, or all of one, or none of one.

## Affective Outcomes

Emotion and affect is strongly related to sport consumption (Moital et al., 2019) and responses such as positive emotions, product engagement, and happiness can be outcomes of consuming sport (Doyle et al., 2016; Jang et al., 2019). Emotional responses can be activated in specific sport consumption settings, such as attending a game live in person (Cho and Lee, 2019), engaging in traditions such as rivalries (Limbach et al., 2019), or following one’s favorite athlete (Chang et al., 2018). As well, as the focus of this paper is, sport fans can activate emotional responses through their connection to other fans in a sport fan community. The affective benefits achieved from membership in a sport fan community are “those relating or arising from influencing feelings or emotions” (Mastromartino et al., 2019b). The Team Identification-Social Psychological Health Model from Wann (2006) shows us that the increase in social connections that comes with identifying as a fan of a team leads to well-being benefits. Building on that, Wann et al. (2011) found that team identification was positively related to a fan’s social psychological health. Similarly, Wann et al. (2008) found that for even lowly identified fans, being connected to the fan community was more of a significant predictor of well-being than attending games. These studies suggest that it is likely an individual’s psychological well-being can increase through an association with the fan community of a sport team.

As a member of a sport fan community, individuals achieve benefits such as boosts in self-esteem and feelings of belonging because they are associated with a group of like-minded individuals and can feel like they are part of an exclusive club (Wann and James, 2019). Sport often brings disparate people together to share a communal experience (Kutcher, 1983; Melnick, 1993) and this shared experience leads to a kinship between members (McAlexander et al., 2002). Through their membership, the value members gain is a sense of uniqueness because a fan community has certain requirements needed for membership (such as knowledge or passion for the brand) that not everyone can obtain (Tian et al., 2001; Wu et al., 2012). The reasons individuals consume sport vary from person to person, but according to Wann (1995), and group affiliation is one of the most common motivations. Beyond having a shared passion for a sport or team, other factors can bring individuals together to create a common group identity such as symbols from associated communities (Heere and James, 2007; Heere et al., 2011), class affirmation (Taylor, 1987), or national unity (Klein, 1984, 1991). For example, McDonald et al. (2002) note that members of exclusive country or sailing clubs can be aligned by social class and then enjoy the benefits of being members in those exclusive clubs. Even though they may be united by other affiliations, it is the membership in the exclusive sport club or community that leads to self-esteem benefits.

When a brand employs an exclusivity marketing strategy, it is an example of a brand hijack Wipperfürth (2005) because it seeks to seduce the consumer by aiming at their heart, not their brain: “Is there anything you want more than the thing you can’t have?”(Wipperfürth, 2005). Further elaborating on that, the author uses the example of Red Bull as a brand that creates an air of exclusivity that attracts members of their fan community. Red Bull focuses their sampling efforts on exclusive sub communities in order to create a deep experience for the consumer. They do not focus on how many people sample the product, for them it’s who and how that matter. As well, Red Bull has limited access and availability of their merchandise. It is only their spokespeople such as celebrities and athletes who get access to t-shirts and hats, consumers do not have the ability to purchase any merchandise. This forces consumers to work hard to obtain any Red Bull related merchandise, and when they do they consider it a prized possession while giving them a story to tell their friends and family. Red Bull’s strategy “encourages consumers to feel special, as though they’ve discovered something different” (Wipperfürth, 2005).

Feelings of uniqueness can be especially heightened if an individual identifies as a member of the community in a niche sport (Mastromartino et al., 2019a; Mastromartino et al., 2020). In a study on fans of surfing, Moutinho et al. (2007) noted that fans in this community displayed similar attitudes and behaviors because it is a very specific sport that takes dedication to understand and be involved with. In this specific fan community, individuals would purchase and wear certain surfing apparel brands to signify they were part of the exclusive surf fan community. No one other than a diehard surf fan would wear these brands, something that draws them closer to the fan community. Another example of this is in an analysis on mountain climbers and rugby players by Donnelly and Young (1988) who noted there is a complex process of identity construction that is built on subculture affiliation, or membership in exclusive groups. Through this previous research, it is clear that being part of an exclusive club with like-minded individuals is desirable for sports consumers and an integral part of a sport fan community, leading to affective benefits through their sense of membership in the community.

For fans to feel part of a fan community, they also need a sense of acceptance for their religious beliefs, gender, race, sexual orientation, or any other important part of their identity outside of their fandom. Not only is tolerance important for fan communities, positivity around those elements, and for welcoming new people into the community is important in feeling connected to that community. An example of this comes from Darling-Wolf (2004) in an examination of identity and gender in a fan community of a Japanese celebrity. The research found that this was a popular community because female fans felt that the community was a safe haven where other members of the community were supportive and not view their fandom any differently because they were a woman. This was an online community with clearly defined rules from the webmaster, but this community collectively acted to be positive and accepting of each other, and resulting in an enjoyable fan community experience for those involved. In a sport consumer context, Cleland (2015) found that British football fan message boards have been increasing in inclusive and tolerant attitudes, specifically through the rejection of homophobia. This has been a common topic of research in football fan communities and has aligned with other studies that show decreasing homophobia has been found among sport fans (Adams, 2011; Cashmore and Cleland, 2012; Nylund, 2012). However, there have been opposite results found in some sport fan communities, such as Kian et al. (2011) who found that homophobic and sexist posts went uncontested on message boards in fan communities of American football. The ability to be accepted for who they are has a strong impact in how a fan can connect with a fan community and the type of benefits they receive from acting in that community.

In addition to the feeling of acceptance from fellow community members, sport fans receive further affective benefits by having a direct relationship with the organization through their membership in the fan community. Although fan communities are often self-sufficient and operate within themselves, a relationship with the organization can give a fan community legitimacy and make members feel like they are part of the team they follow. In business practices, this function is often referred to as relationship marketing which can be defined as “all marketing activities directed toward establishing, developing, and maintaining successful relational exchanges” (Morgan and Hunt, 1994, p. 22). When it comes to relationship marketing in the sport fan context, professional sport organizations want their fans to feel like they contribute to the team and with that requires being interactive with the fan community. This interaction is a two-way process and (Bühler and Nufer, 2010) outline some examples of sport organizations that have a direct relationship with their fans. One is the New Zealand All Blacks national rugby team who, through their website, invite fans to send messages to the players, and then post those messages through the team’s locker room. Some sport organizations have official fan clubs where in return for their membership; fans receive perks such as gifts from the team and communication with players. An example of this is Juventus of the Italian Seria A football league who charges $12 per year for fans to join and in return the fans receive autographed player photos, access to their online fan community, and an official team e-mail address. There are variations of these types of membership loyalty programs, but all serve to highlight the affective benefits from having a direct relationship with the organization.

Bee and Kahle (2006) examined why having a relationship with the organization is important for sport consumers as well. Their research argues that many sport organizations use market data to analyze simple purchasing behavior information such as the amount paid, the type of product purchased, where the product was purchased, and if it was a repeat purchase. However, with relationship marketing, an organization can work to build a stronger emotional connection with their fan community by approaching it in a way that allows them to understand why a particular product was purchased and assess the likelihood of a repeat purchase. This suggests that sport organizations should view their fans as lifetime partners and work to understand their changing needs, instead of a strong focus on short-term transactions and immediate profits (Stavros et al., 2008). Further research shows that the length of a relationship between the consumer and organization can lead to an increased sense of loyalty. Research findings by Raimondo et al. (2008) tell us that the longer someone feels they are loyal to a certain brand, the more likely they are to have positive attitudes such as satisfaction and trust, and as well as increased consumer behavior. Practically speaking, this research suggests sport organization should focus on length of a time as a fan in order to develop social equity with their fan base.

## Conclusion

The concept of fan communities is a growing and evolving topic in sport research and among sport practitioners. Currently, most investigations on the affective outcomes of membership in a sport fan community are conceptual in nature and more empirical evidence needs to be discovered and disseminated in order for sport organizations to understand their consumer base and tailor marketing strategies around the emotions of fans. As well, future research should consider other sociodemographic factors such as age, income, race, gender, and sexuality which may mediate the affective outcomes of achieving a sense of membership in a sport fan community. This current work focuses on the affective outcomes on the individual fan level, but future research should also consider these outcomes on the group and societal level. Previous conceptualizations have suggested that affective benefits exist through membership in a sport fan community, but future research needs to dig deeper to fully understand all the factors at play. Understanding sport fans is continually an evolving process as technology and society changes and continued observation of their behavior in fan communities is essential for sport academics and practitioners in order to remain at the cutting edge of research and practice.

## Author Contributions

BM wrote the first draft of the manuscript based on BM and JZ’s collective conceptualization. BM and JZ edited subsequent drafts.

## Conflict of Interest

The authors declare that the research was conducted in the absence of any commercial or financial relationships that could be construed as a potential conflict of interest.
